# NiFe Alloy Integrated with Amorphous/Crystalline NiFe Oxide as an Electrocatalyst for Alkaline Hydrogen and Oxygen Evolution Reactions

**DOI:** 10.1021/acsomega.3c00322

**Published:** 2023-03-29

**Authors:** Guoyu Shi, Chisato Arata, Donald A. Tryk, Tetsuro Tano, Miho Yamaguchi, Akihiro Iiyama, Makoto Uchida, Kazuo Iida, Sumitaka Watanabe, Katsuyoshi Kakinuma

**Affiliations:** § Hydrogen and Fuel Cell Nanomaterials Center, University of Yamanashi, Miyamae 6-43, Kofu 400-0021, Yamanashi Japan; ∥ R&D Center, Nihon Kagaku Sangyo Co., Ltd., Nakane 1-28-13, Soka, Saitama 340-0005, Japan

## Abstract

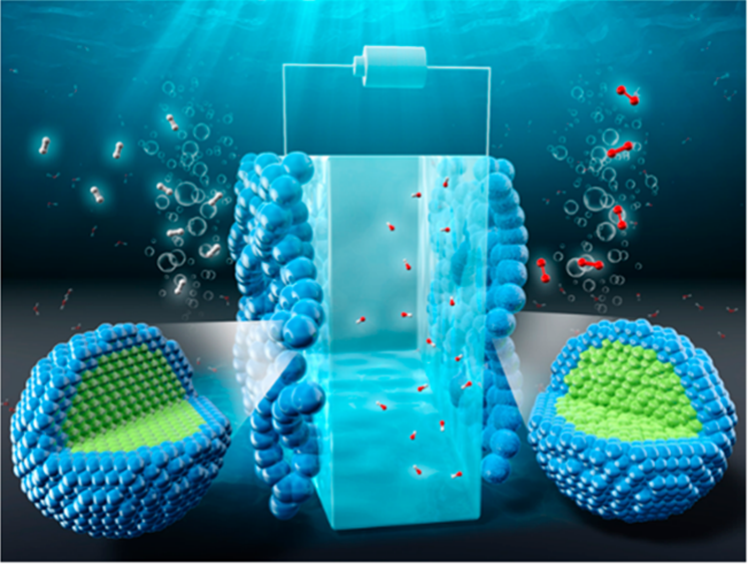

The rational design of efficient and low-cost electrocatalysts based on earth-abundant materials is imperative for large-scale production of hydrogen by water electrolysis. Here we present a strategy to prepare highly active catalyst materials through modifying the crystallinity of the surface/interface of strongly coupled transition metal–metal oxides. We have thermally activated the catalysts to construct amorphous/crystalline Ni–Fe oxide interfaced with a conductive Ni–Fe alloy and systematically investigated their electrocatalytic performance toward the hydrogen evolution and oxygen evolution reactions (HER and OER) in alkaline solution. It was found that the Ni–Fe/oxide material with a crystalline surface oxide phase showed remarkably superior HER activity in comparison with its amorphous or poorly crystalline counterpart. In contrast, interestingly, the amorphous/poorly crystalline oxide significantly facilitated the OER activity in comparison with the more crystalline counterpart. On one hand, the higher HER activity can be ascribed to a favorable platform for water dissociation and H–H bond formation, enabled by the unique crystalline metal/oxide structure. On the other hand, the enhanced OER catalysis on the amorphous Ni–Fe oxide surfaces can be attributed to the facile activation to form the active oxyhydroxides under OER conditions. Both are explained based on density functional theory calculations. These results thus shed light onto the role of crystallinity in the HER and OER catalysis on heterostructured Ni–Fe/oxide catalysts and provide guidance for the design of new catalysts for efficient water electrolysis.

## Introduction

In response to environmental pollution issues, the use of renewable energy replacing fossil fuels has increased over the past few decades. However, reliable energy storage systems are required to compensate for the intermittent nature of energy generation from renewable energy sources, such as solar and wind power. Water electrolyzers show great promise for solving this problem by converting the surplus renewable energy power into chemical energy stored in H_2_ chemical bonds.^1−4^ The H_2_ can be used in fuel cells to produce electricity to power electric vehicles and residential systems. Water electrolysis for H_2_ production has been conducted under both acidic or alkaline conditions. Alkaline water electrolysis allows the use of nonprecious metal catalysts for the hydrogen evolution reaction (HER) and the oxygen evolution reaction (OER), so the cost of catalysts is lower compared with those for proton-exchange membrane (PEM)-based water electrolysis.^4−8^ Ni-based materials are widely used as the electrodes or electrocatalysts in alkaline electrolysis because of their high alkaline stability and high electrocatalytic activity for both the HER and OER. Ni alloys, such as NiMo,^9^ NiCo,^10^ and NiFe,^11^ were found to show high activity and stability for the alkaline HER. The Ni-based oxides and hydroxides have been extensively investigated as OER catalysts in alkaline media. Integrating metals with oxides/hydroxides can lead to promising catalysts to further promote the HER and OER performance. A Ni/NiO heterostructure-based catalyst was found to exhibit superior HER activity compared to Ni itself, which was proposed to be due to the presence of synergistically active sites involving meta-oxide interfaces for HER catalysis.^12^ Recently, it was reported that interfacial electron rearrangement favorably modulated the electronic structure of Ni–Ni(OH)_2_, which facilitated the HER kinetics.^13^ Xu et al. found NiCo@NiCoO_2_ core@shell nanoparticles showed a high OER activity due to large specific surface areas, high conductivity, and multiple electrocatalytic active sites.^14^ It was also found that the NiFe metal–organic framework (MOF) supported on graphene-nanoplatelets could be used as a superior and ultradurable (>1000 h) anode for alkaline water electrolysis.^15^ In addition, tuning the phase structure of these materials is expected to provide exciting possibilities to modify their chemical properties and thus optimize their electrocatalytic performance. A previous study has demonstrated that NiFe hydroxide nanoparticles exhibiting low crystallinity and Fe-incorporation-induced charge transfer showed excellent OER performance.^16^ Cai et al. reported that an amorphous NiFe alloy exhibited high-performance toward OER catalysis due to the short-range ordering of the amorphous structure promoting the exposure of active sites.^17^ Meena et al. reported an active mesoporous Ni_2_P@FePO_*x*_H_*y*_ catalyst containing crystalline Ni_2_P and amorphous FePO_*x*_H_*y*_ phases with more electrocatalytic active sites showed OER overpotential of 360 mV at a current density of 1 A cm^–2^ in 1 M KOH with long-term durability (12 days).^18^ Similarly, an amorphous NiFeOOH catalyst on surface-activated carbon fiber paper (CFP) was found to be highly active and stable when utilized as the anode of an alkaline anion exchange membrane water electrolyzer.^19^ Meena et al. also prepared a self-supported catalyst via direct growth of oxovanadate-doped cobalt carbonate (VCoCO_*x*_@NF) on nickel foam (NF), which demonstrated high activity for both HER and OER in alkaline media.^20^ However, to the best of our knowledge, the effect of amorphization or crystallinity of alloy-oxide heterostructures (expected to take advantage of both the conductive and catalytically active phases/interfaces) on both the HER and OER catalysis in alkaline media has yet to be investigated.

Herein, we report new electrocatalysts consisting of NiFe alloys and their oxides with heterointerfaces and tuned crystallinity for catalyzing both the HER and OER in alkaline solution. The crystallinity of the oxide phases can be tuned by regulating the thermal annealing conditions without changing the chemical composition of the catalyst. This work aims to develop efficient NiFe/oxide catalysts for HER and OER through tuning structural crystallinity and elucidate the structure–property relationships of the catalysts by a combined experimental and density functional theory (DFT) study, which will play an important role in new catalyst design for alkaline water electrolysis applications.

## Experimental Section

The nanostructured NiFe oxide materials were prepared using a scalable flame oxide-synthesis method.^21,22^ Nickel and iron octoates were mixed at the desired mole ratio and then dissolved in turpentine oil, followed by magnetic stirring at room temperature for 30 min. The solution was sent to an atomizer to be directly injected into a flame (temperature >1600 °C) produced by the combustion of propane (1 L min^–1^) with oxygen (5 L min^–1^). The resulting brown powder was collected with a high-efficiency particulate air filter. The as-prepared sample (8 g) was reduced with 100% H_2_ at 500 °C for 1 h. A second portion (50 g) sample was reduced with 100% H_2_ at 450 °C for 1 h. Finally, the resulting products were treated with 20% O_2_/N_2_ at room temperature for 30 min. Because the first sample underwent a higher temperature H_2_ reduction, the oxide phase would be decreased or presumably more disordered compared with the second sample. The two products thus obtained were tentatively denoted as NiFeO-1 and NiFeO-2, respectively.

The morphology and element distribution of the obtained NiFe oxide catalysts were observed with transmission electron microscopy (TEM, H9500, Hitachi High-Tech Co., Japan) and scanning transmission electron microscopy (STEM, HD-2700, Hitachi High-Tech Co., Japan) equipped with an energy-dispersive X-ray spectrometer (EDX, Quantax XFlash 5010, Bruker AXS GmbH, Germany). X-ray diffraction (XRD) patterns were recorded using an X-ray diffractometer with Cu Kα radiation (0.15406 nm, 40 kV, 40 mA, Ultima IV, Rigaku Co., Japan). X-ray photoelectron spectra (XPS) were collected on an X-ray photoelectron spectrometer using Mg Kα radiation (JPS-9010, JEOL Ltd. Japan). The obtained spectra were analyzed with the JEOL SpecSurf software package to employ Shirley background subtraction and Gauss–Lorentz peak fitting. The binding energies (BE) were calibrated using the Au 4f_7/2_ peak (BE = 83.3 eV) of a gold wire as a reference.

The electrochemical measurements were performed using a rotating disk electrode (RDE) setup with a HZ-5000 potentiostat (Hokuto Denko Co., Japan).^21−25^ The catalysts were coated onto a glassy carbon (GC) substrate (diameter 5 mm, Hokuto Denko Co., Japan) with a constant loading of 40 μg cm^–2^. An aliquot of 0.2 wt % Nafion (diluted with ethanol and water at 3:2 vol %) solution was pipetted onto the dried catalyst layer to yield a film thickness of 0.05 μm. The thickness of the Nafion film was calculated based on its mass and the electrode surface area assuming a density of 1.98 g cm^–3^ in its dry state. A gold wire and a reversible hydrogen electrode (RHE) were employed as the counter electrode (CE) and reference electrode, respectively. All electrochemical measurements were conducted in 1 M KOH (Kanto Chemical Co., Inc.), which had been purified in advance based on a pre-electrolysis method.^26^ Polarization curves for HER and OER were recorded at 10 mV s^–1^ and 2500 rpm. The *iR*-loss (ohmic drop) was excluded from the electrode potential by measuring the ohmic resistance of the electrolyte solution at the open circuit voltage (OCV) with a potentiostat equipped with an AC impedance analyzer (PGSTAT302N, Metrohm Autolab B. V., The Netherlands). For comparison, commercial Pt/C (46.3 wt %, TEC10E50E, Tanaka Kikinzoku Kogyo K.K.) and IrO_2_ (Tokuriki Honten Co. Ltd.) powder catalysts were also tested (Pt and IrO_2_ loadings on the electrode were 5 and 40 μg cm^–2^, respectively).

In situ Raman spectra were obtained with a Senterra Raman microscope (Bruker Corp.) with the excitation laser light at 532 nm and a 50× magnification objective. Automatic baseline correction was conducted using the “rubber band” method. Electrochemical measurements were performed using a Raman electrochemical flow cell (ECFC, Redoxme AB, Sweden) with an Ag/AgCl reference electrode, a Pt wire as a counter electrode, and an Au substrate coated with catalyst as a working electrode. The electrolyte was purified 1 M KOH. A HZ-5000 potentiostat was used to control the potential. The measured potentials vs Ag/AgCl were converted to the RHE scale.

DFT calculations were carried out by use of the DMol^3^ package (Materials Studio, version 2021, BIOVIA Co., USA). Details of the calculation procedures can be found in the Supporting Information.

## Results and Discussion

**Figure 1:**
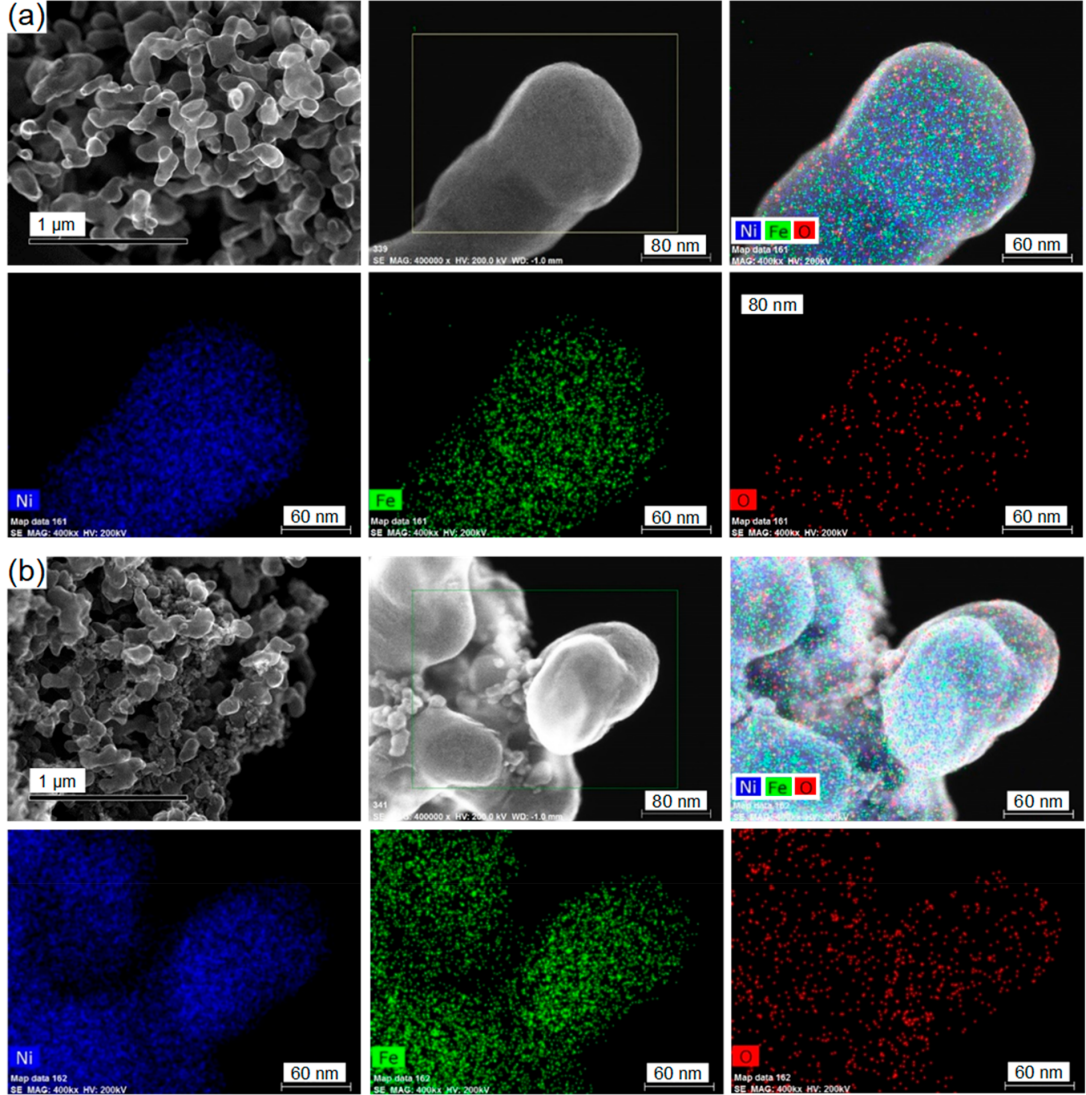
STEM-EDX mapping images of (a) NiFeO-1 and (b) NiFeO-2 catalysts.

Figure 1 shows STEM-EDX mapping images of the NiFeO-1 and NiFeO-2 catalysts. The STEM images clearly show that the NiFeO-1 (Figure 1a) was mainly composed of interconnected particles with uniform distribution, while the particles of NiFeO-2 (Figure 1b) were not as uniform as NiFeO-1. From the EDX mapping images, Ni, Fe, and O were generally seen to be uniformly distributed within the catalyst. The mapping results yielded identical Ni/Fe atomic ratios of 0.88:0.12 (Table S1) for the two catalysts, and considering the basic structure of NiO (Fe as dopant), we have denoted the two catalysts as Ni_0.88_Fe_0.12_O-1 and Ni_0.88_Fe_0.12_O-2 hereinafter.

**Figure 2:**
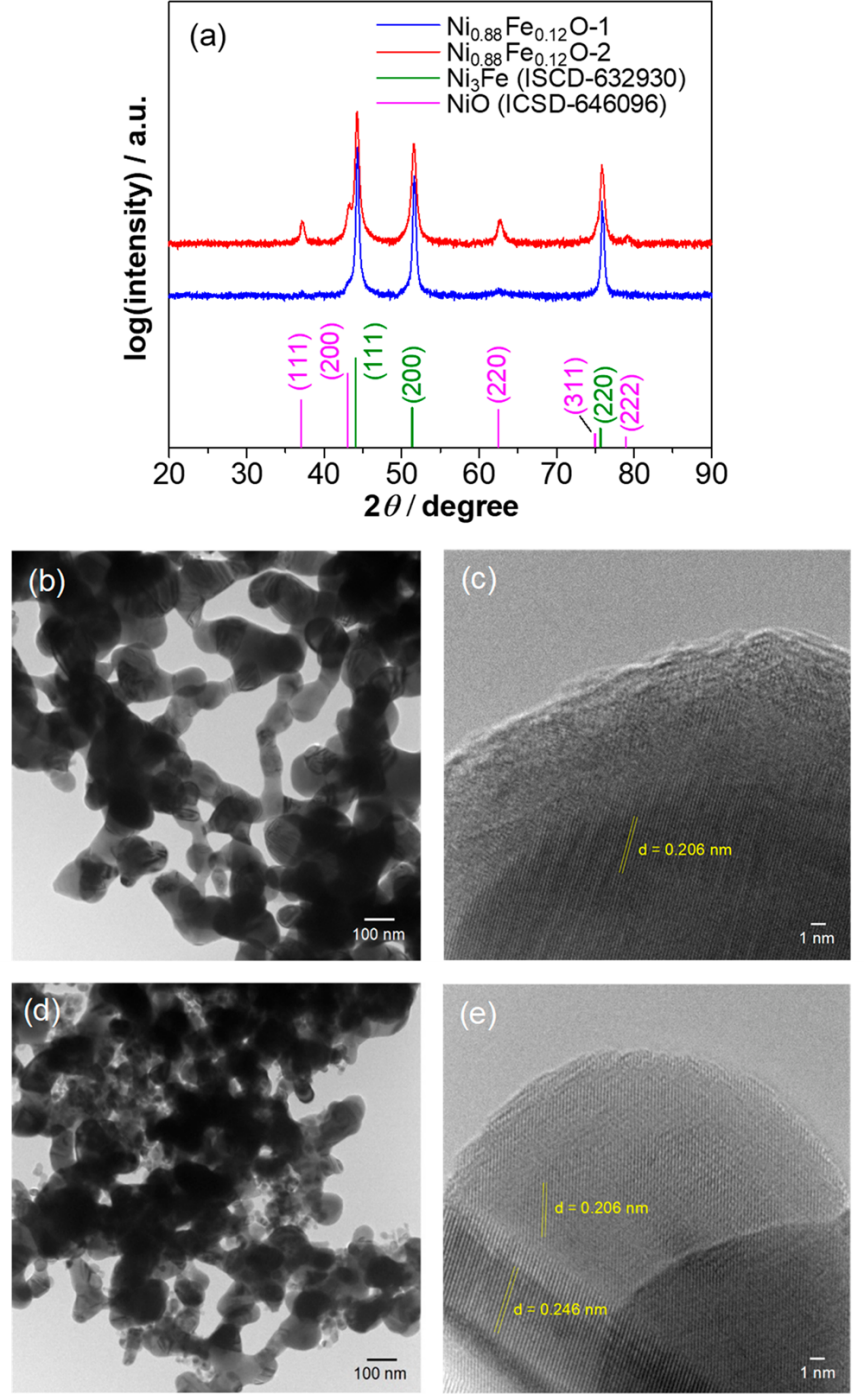
(a) Powder XRD patterns of the two Ni_0.88_Fe_0.12_O catalysts. (b, c) TEM and high-resolution (HR) TEM images of Ni_0.88_Fe_0.12_O-1 catalyst. (d, e) TEM and HRTEM images of Ni_0.88_Fe_0.12_O-2 catalyst.

**Figure 3:**
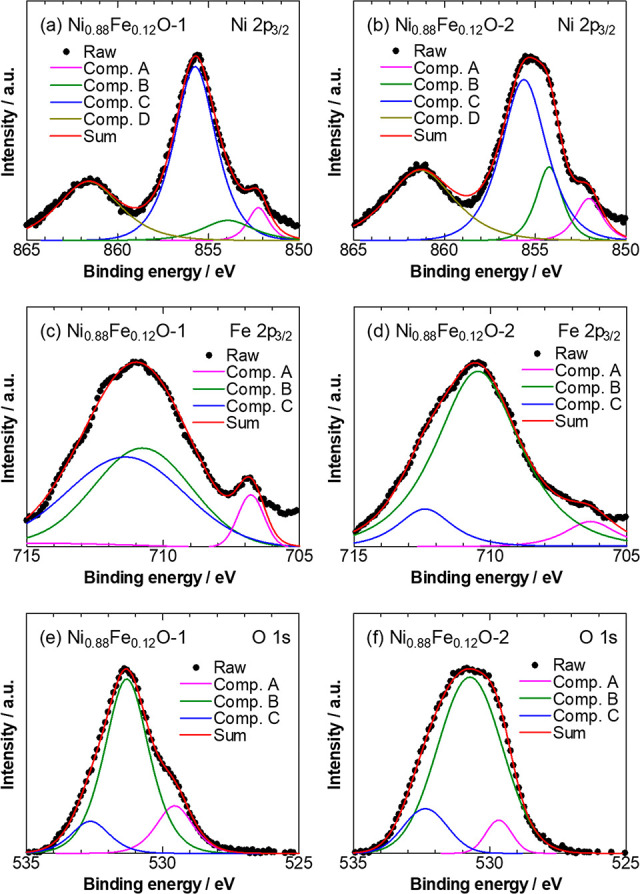
XPS analysis of Ni_0.88_Fe_0.12_O-1 (a, c, e) and Ni_0.88_Fe_0.12_O-2 (b, d, f) catalysts including the fit from individual component contributions.

The XRD patterns of the two Ni–Fe-based oxide catalysts are shown in Figure 2a. Based on the reference patterns, the typical diffraction peaks assigned to (111), (200), and (220) planes of the face-centered cubic (FCC) structure of Ni–Fe alloy crystallites have been identified for both catalysts. No intense peaks corresponding to oxide phases were observed for Ni_0.88_Fe_0.12_O-1, suggesting that most of the oxides were poorly crystallized or existed in an amorphous state. In contrast, for Ni_0.8_Fe_0.2_O-2, clear diffraction peaks ascribed to the NiO phase or Ni–Fe–O solid solution dominated by Ni appeared, indicating the high crystallinity of the oxide phases. Consistent with the STEM images in Figure 1, the TEM images (Figures 2b,d) also reveal an interconnected network structure for the two catalysts. The high-resolution (HR) TEM image of Ni_0.88_Fe_0.12_O-1 (Figure 2c) shows clear lattice fringes with spacing of 0.206 nm for the inner part, which is assigned to the (111) plane of the Ni–Fe alloy FCC crystal. The outer surface layers exhibited an amorphous characteristic without clearly resolved lattice fringes and are mainly composed of Ni–Fe oxide, as revealed from the followed XPS analysis (Figure 3). The structure of the amorphous surface and crystalline inner part was also supported by the diffused rings and bright spots observed in the corresponding selected-region fast Fourier transform (FFT) patterns (Figure S1). In contrast to Ni_0.88_Co_0.12_O-1 possessing amorphous/low-crystalline surfaces covered on a highly crystallized phase, the Ni_0.88_Fe_0.12_O-2 was seen to be highly crystallized from the bulk to the surface (Figure 2e). The interplanar distances of 0.206 and 0.246 nm correspond to (111) plane of FCC Ni–Fe and Ni–Fe–O, respectively. The oxide was shown to extend from the interface with the alloy to the outer surface. The XPS analysis was also conducted to provide the surface-specific chemical information for the catalyst particles. The spectra shown in Figure 3 confirmed the involvement of Ni, Fe, and O elements in the surfaces of the two catalysts. In the Ni 2p_3/2_ spectra (Figure 3a), Ni_0.88_Fe_0.12_O-1 showed typical peaks for Ni^2+^ of NiO (component B) and corresponding shakeup satellite (component D).^27^ The peak located at 855.6 eV (component C) might be assigned to Ni(OH)_2_ or Ni^3+^ species, e.g., Ni_2_O_3_ and NiOOH.^28,29^ An additional shoulder peak was seen at 852.5 eV (component A), corresponding to metallic Ni^0^.^30^ As compared with Ni_0.88_Fe_0.12_O-2 (Figure 3b), Ni_0.88_Fe_0.12_O-1 exhibited a higher peak area ratio for component C, indicating that there might be more OH groups existing on the surface of Ni_0.88_Fe_0.12_O-1 or the degree of surface oxidation might be higher for Ni_0.88_Fe_0.12_O-1, consistent with the amorphous oxide surface structure, as reported earlier.^17^ In the Fe 2p_3/2_ spectra (Figures 3c,d), the peaks for components B and C can be assigned to Fe^2+^ and Fe^3+^, respectively. The existence of metallic Fe was also confirmed, as seen from the shoulder peak for Fe^0^ at 705–706 eV (component A).^31^ Thus far, the Ni and Fe 2p_3/2_ spectra of Ni_0.88_Fe_0.12_O-1 and Ni_0.88_Fe_0.12_O-2 have demonstrated that Ni and Fe were mainly present in the oxidized states on the surface, coexisting with a small amount of metallic Ni and Fe. In Figures 3e,f, the O 1s spectra were shown to be fitted well with three components: the component A is attributed to the characteristic lattice oxygen bonding to Ni^2+^ in NiO; the component B could be contributed from surface hydroxyl groups; and the component C could be assigned to adsorbed water and/or chemisorbed oxygen.^29,30,32^ Importantly, for Ni_0.88_Fe_0.12_O-1, the peak for component B shifted to a higher binding energy by approximately 0.7 eV compared with that for Ni_0.88_Fe_0.12_O-2, which could be related to the presence of oxygen vacancies or defect sites with low-coordinated oxygen caused by structural amorphization or disorder at the surface of Ni_0.88_Fe_0.12_O-1 (see Figure 2c).^29,30,33,34^ Therefore, based on the above results, we have demonstrated the presence of oxidized Ni–Fe species on the surfaces of Ni_0.88_Fe_0.12_O-1 and Ni_0.88_Fe_0.12_O-2. The amorphous Ni–Fe oxide surface of Ni_0.88_Fe_0.12_O-1 might be correlated with increased vacancy defects or low-coordinated sites in comparison with the more crystalline Ni_0.88_Fe_0.12_O-2.

**Figure 4:**
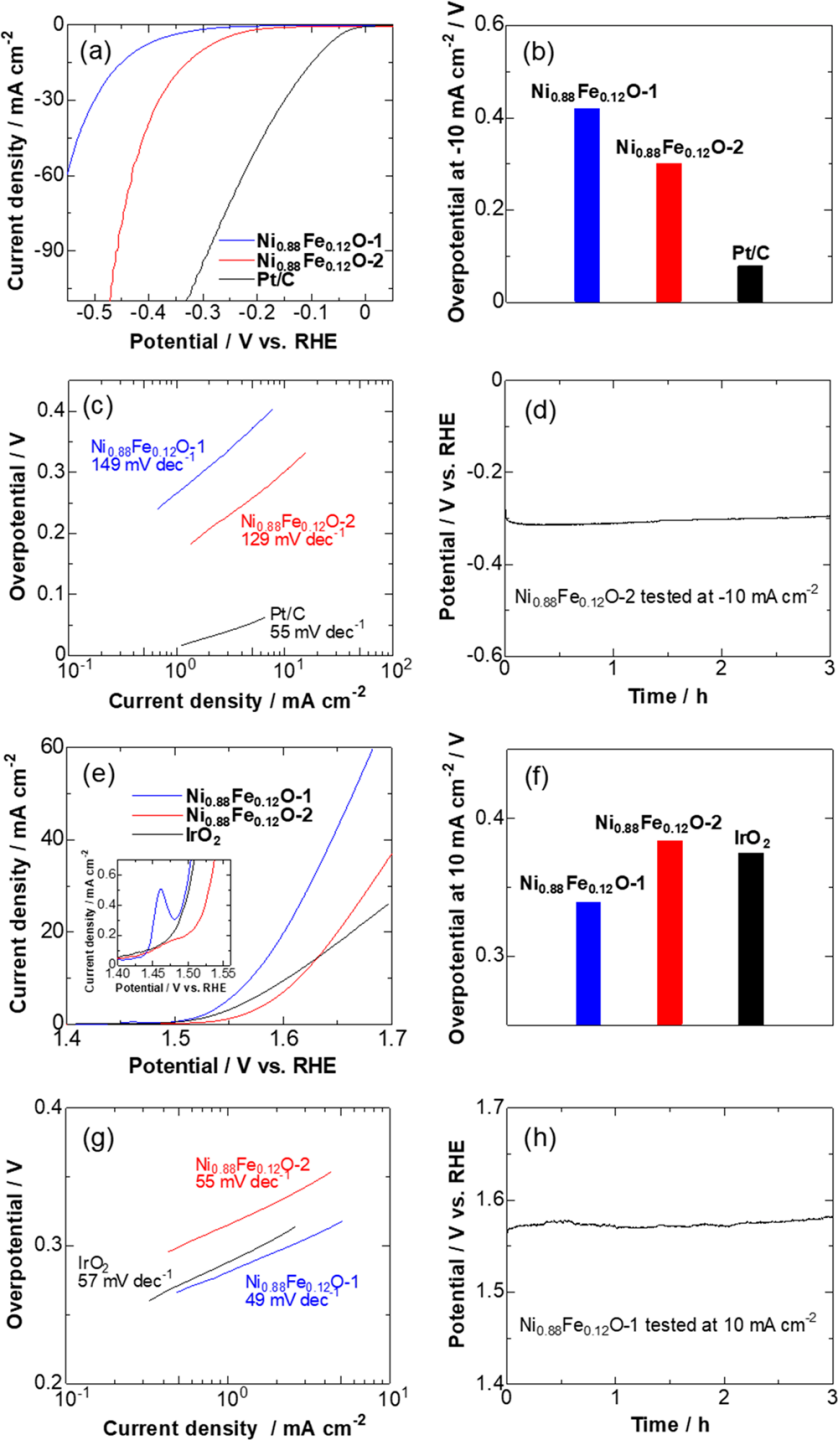
(a) Polarization curves for HER in 1 M KOH (10 mV s^–1^, 2500 rpm). (b) Overpotentials for HER at −10 mA cm^–2^. (c) Corresponding Tafel plots. (d) Potential–time curve of Ni_0.88_Fe_0.12_O-2 for HER stability at −10 mA cm^–2^. (e) Polarization curves for OER in 1 M KOH (10 mV s^–1^, 2500 rpm) with the inset showing an enlargement of the low potential region. (f) Overpotentials for OER at 10 mA cm^–2^. (g) Corresponding Tafel plots. (h) Potential–time curve of Ni_0.88_Fe_0.12_O-1 for OER stability at 10 mA cm^–2^.

Figures 4a shows the HER polarization curves in 1 M KOH for Ni_0.88_Fe_0.12_O-1, Ni_0.88_Fe_0.12_O-2, and commercial Pt/C catalysts. It is apparent that, despite being less active than Pt/C, the Ni_0.88_Fe_0.12_O-2 catalyst displayed much better HER activity in comparison to Ni_0.88_Fe_0.12_O-1, reaching a current density of −10 mA cm^–2^ at an overpotential of 300 mV, which was 120 mV less than that of Ni_0.88_Fe_0.12_O-1 (Figure 4b). The Pt/C catalyst exhibited near-zero onset potential and required an overpotential of only 78 mV to reach −10 mA cm^–2^, demonstrating its preeminent HER activity. The current has been also normalized by the electrochemically active surface area (ECSA) of the catalyst estimated from the double-layer capacitance in the cyclic voltammetry curves (Figure S2) in order to provide specific or intrinsic activities. From the ECSA-normalized HER polarization curves, the specific activities of Ni_0.88_Fe_0.12_O-2 at given potentials are shown to be significantly higher than those of Ni_0.88_Fe_0.12_O-1 (Figure S3a), indicating that the catalytic sites of Ni_0.88_Fe_0.12_O-2 were intrinsically more active toward HER. The measured Tafel slope of Ni_0.88_Fe_0.12_O-2 was 129 mV dec^–1^, as presented in Figure 4c, which was apparently larger than that of the Pt/C catalyst (55 mV dec^–1^) but lower than that of Ni_0.88_Fe_0.12_O-1 (149 mV dec^–1^), demonstrating faster HER kinetics of Ni_0.88_Fe_0.12_O-2 compared with Ni_0.88_Fe_0.12_O-1, with the HER process possibly following the Volmer–Heyrovsky mechanism.^35^ These results indicate that the crystalline Ni–Fe oxide structure integrated with Ni–Fe alloy in Ni_0.88_Fe_0.12_O-2 is favorable for enhancing the HER catalysis. On the other hand, a decrease in crystallinity of the oxide would result in a lower HER activity, as seen for Ni_0.88_Fe_0.12_O-1. Given the superior HER activity of Ni_0.88_Fe_0.12_O-2, its electrocatalytic stability was further evaluated by holding the electrode at a constant current density of −10 mA cm^–2^. As shown in Figure 4d, the Ni_0.88_Fe_0.12_O-2 exhibited high stability with negligible change in the potential after 3 h of HER catalysis.

The electrocatalytic activity toward the OER in 1 M KOH was also investigated, and the polarization curves are shown in Figure 4e. It is evident that the Ni_0.88_Fe_0.12_O-1 catalyst significantly outperforms Ni_0.88_Fe_0.12_O-2 and commercial IrO_2_ catalysts over the whole potential range. Interestingly, contrary to the HER activity trend, Ni_0.88_Fe_0.12_O-1 exhibited superior OER specific activity (per ECSA) in comparison with that for Ni_0.88_Fe_0.12_O-2 (Figure S3b). The enhanced OER activity could be ascribed to the presence of amorphous oxides at the surface of Ni_0.88_Fe_0.12_O-1, facilitating an enrichment of the NiOOH active species, as revealed from the remarkable oxidation peak for NiOOH formation at 1.46 V vs RHE (see the inset of Figure 4e). In alkaline solution, hydroxides are present at the surface of Ni oxides and will be transformed/oxidized to NiOOH via a reaction of Ni(OH)_2_ + OH^–^ → NiOOH + H_2_O + e^–^ under OER conditions.^28,36^ The amorphous oxide structure (Ni_0.88_Fe_0.12_O-1) would be favorable for such a transformation, while it might be suppressed upon the presence of crystalline Ni–Fe–O (Ni_0.88_Fe_0.12_O-2), as indicated from the inset of Figure 4e. The detailed mechanism will be discussed in the DFT section. It is noteworthy that the Ni_0.88_Fe_0.18_O-1 achieved 10 mA cm^–2^_geo_ with an overpotential of 0.34 V (Figure 4f), which is among the best OER performances reported in the literature (0.33–0.50 V).^37^ Moreover, in Figure 4g, Ni_0.88_Fe_0.18_O-1 showed a lower Tafel slope (49 mV dec^–1^) compared with Ni_0.88_Fe_0.18_O-1 (55 mV dec^–1^) and IrO_2_ (57 mV dec^–1^), implying a faster OER kinetics with a chemical rate-determining step possibly involving OH rearrangement via a surface reaction.^38^ Thus, contrary to HER catalysis, the amorphous oxides for Ni_0.88_Fe_0.12_O-1 greatly outperformed their crystalline counterpart, Ni_0.88_Fe_0.12_O-2, for the electrocatalytic OER. Figure S4 shows postanalysis of the Ni_0.88_Fe_0.12_O-1 catalyst after the OER test. It was observed that the crystalline interior and amorphous surface structure were still well maintained, implying the structural stability of the Ni_0.88_Fe_0.12_O-1 material during the OER. We further examined the OER stability of the active Ni_0.88_Fe_0.12_O-1 catalyst at a constant current density of 10 mA cm^–2^. The curve in Figure 4h depicts only a tiny potential increase (1.8% of the initial value) after continuous OER catalysis for 3 h, revealing the high stability of Ni_0.88_Fe_0.12_O-1 to maintain a high OER current density. Thus, we have, for the first time, demonstrated that the crystalline NiFe oxide phase was favorable for the alkaline HER while its amorphous counterpart afforded advantages when catalyzing the alkaline OER. The outstanding HER and OER activities and stabilities of crystalline Ni_0.88_Fe_0.12_O-2 and amorphous Ni_0.88_Fe_0.12_O-1 make them very promising for practical application as noble-metal-free cathodes and anode catalysts in alkaline water electrolyzers.

**Figure 5:**
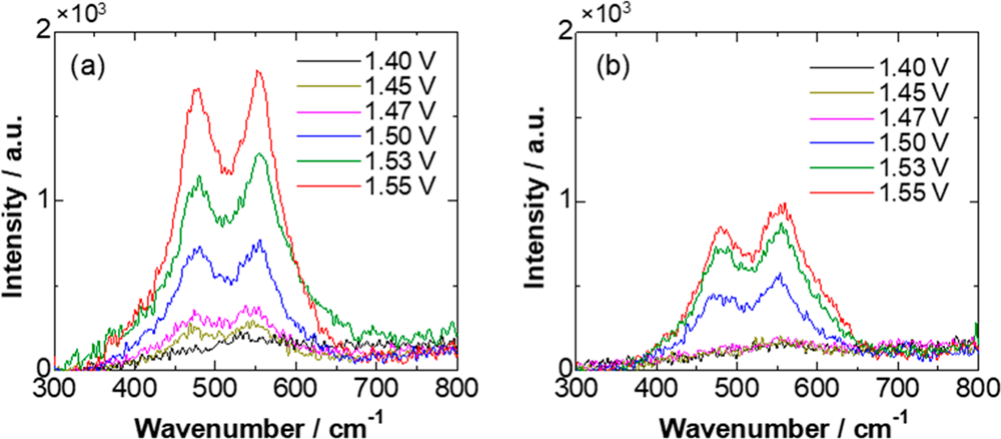
In situ Raman spectra of (a) Ni_0.88_Fe_0.12_O-1 and (b) Ni_0.88_Fe_0.12_O-2 as a function of potential.

To probe the reactive species or catalytically active sites of the Ni–Fe oxides under OER conditions, in situ Raman measurement was carried out in an electrochemical cell. The spectra acquired at varied potentials are displayed in Figure 5. For both catalysts, double peaks were clearly observed at higher potentials, in which the first peak at ca. 475 cm^–1^ and the second peak at ca. 555 cm^–1^ correspond to the E_g_–Ni–O bending vibration and the A_1g_–Ni–O stretching vibration, respectively, which can indicate the presence of NiOOH.^28,36^ For Ni_0.88_Fe_0.12_O-1 (Figure 5a), this pair of peaks started to appear at 1.45 V, which was consistent with the appreciable prepeak of OER (see the inset of Figure 4e). The two vibrations were shown to be still weak even at 1.47 V for Ni_0.88_Fe_0.12_O-2 (Figure 5b), in accordance with the weak prepeak in the OER. At each potential where OER largely occurs (≥1.5 V), the peak intensity for Ni_0.88_Fe_0.12_O-1 was apparently higher than that for Ni_0.88_Fe_0.12_O-2. Thus, the superior OER activity of Ni_0.88_Fe_0.12_O-1 compared to Ni_0.88_Fe_0.12_O-2 was clearly correlated with the increased formation of NiOOH species under anodizing conditions, which could be promoted by structural disorder or amorphization of the oxide phases.

**Figure 6:**
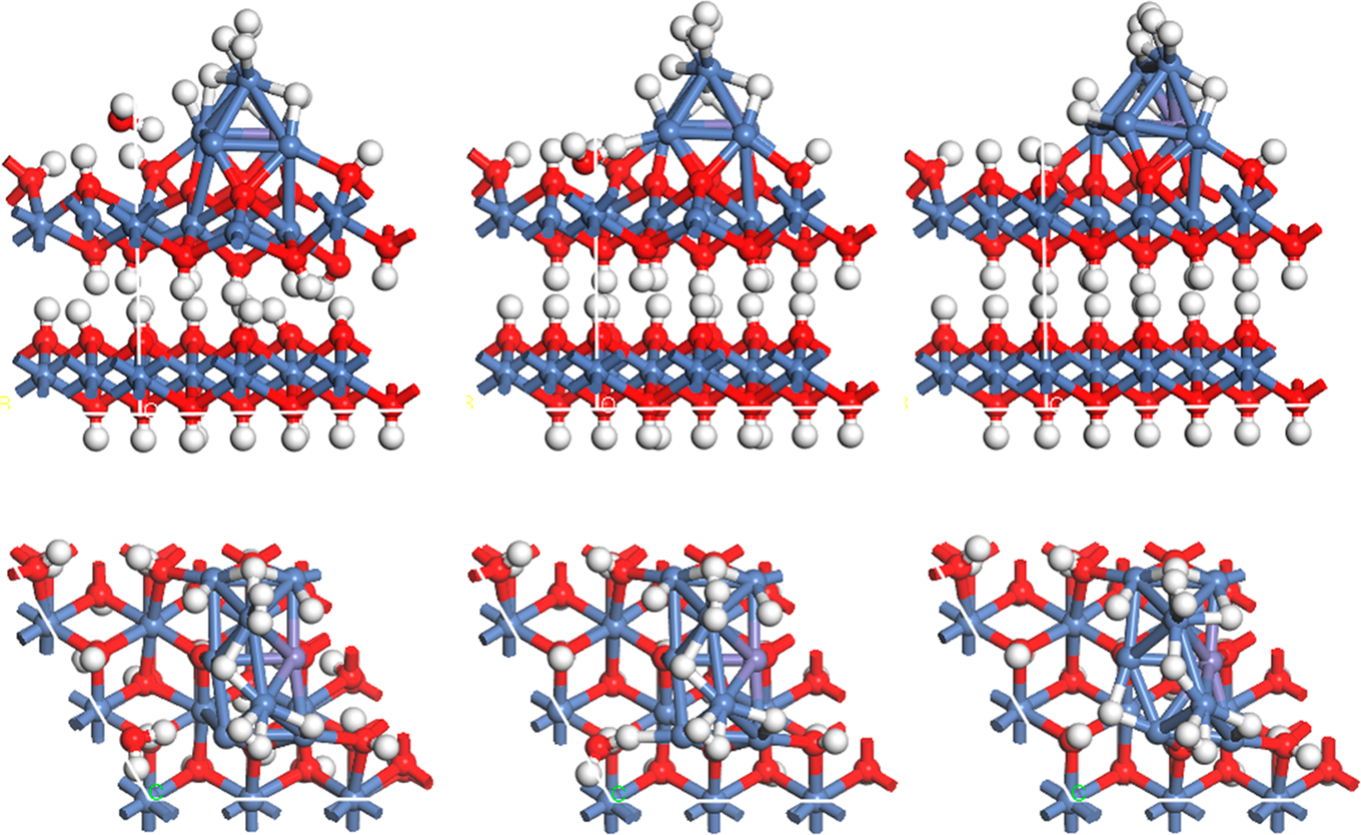
Proposed model for the HER-active surface, with an 8-atom Ni_7_Fe (110) analog metal cluster on a two-layer Ni(OH)_2_ surface, depicting the reverse spillover mechanism. The metal cluster has 9 H atoms initially adsorbed, including two pairs of adsorbed H_2_ at the top Ni sites. The sequence goes from left to right: at the beginning of the reaction, a water molecule adsorbed in an oxygen vacancy is close to the edge of the metal cluster (left), then, a hydrogen atom is in the process of being adsorbed at the corner of the cluster (center), and finally, it has been fully adsorbed on the cluster (right); upper row side view; lower row, top view.

In order to reveal the underlying mechanism of the HER and OER catalysis on the amorphous/crystalline Ni–Fe oxide catalysts, DFT calculations were conducted. To simulate the reduced state, we have employed a two-layer model based on Ni(OH)_2_. To this support, we have added an 8-atom cluster including 7 Ni atoms and 1 Fe atom that is a minimalistic (110) surface, as this surface has been shown to be the most active for water dissociation.^39^ As seen in Figure 6, the Ni_7_Fe cluster has a nearly epitaxial relationship to the Ni(OH)_2_ surface. This ensures a good electronic contact as well as good adhesion. Although there was some relaxation of the bulklike Ni FCC structure, the surface still closely resembles a prototype (110) structure. The (110) surface is thought to operate in a manner similar to that of Pt(110), which is the most active surface of Pt.^40^ In that model, the H_2_ molecule forms easily due to the close proximity of two adsorbed H atoms on single ridge Pt atoms. In the Ni_7_Fe cluster, two pairs of H atoms are shown to be adsorbed at single Ni atoms, with the H–H distances even shorter than they are when adsorbed on Pt(110) and thus quite close to the H–H distance in gaseous H_2_. It is not completely clear why the H atoms are so close together, but the directionality of the bonding on Pt surfaces is known to be strong due to the nature of the d_z2_ orbital, which forces the Pt–Pt–H bond to be more linear.^41^ The weaker directionality on the Ni metal surface allows the Ni–Ni–H angle to be less linear and thus the H–H-distance to be smaller. The desorption of H_2_ from the surface was found to be overall energetically favorable, with an energy change of ca. −0.4 eV. The activation energy for that process is expected to be relatively small in comparison with that for the adsorption of an H atom from an adsorbed water molecule, as shown in Figure 6, which is estimated to be in the range from 0.2 to 0.4 eV and thus to be the rate-determining step in the reaction.

This reaction is considered to be a kind of “reverse spillover” process, which has been proposed as a possible mechanism for the HER.^42−44^ The well-known spillover reaction involves H_2_ dissociation on a metal particle, with the adsorbed H atoms then being transported to a metal oxide support surface, exemplified by Pt particles adsorbed WO_3_.^45^ Several recent papers have described the synergy between Ni and NiO in the HER.^46−48^ In particular, Zhao et al. have shown how the mechanism would work, with the Volmer step (adsorption of H) on the NiO followed by the transport of H to the Ni surface, where 2 H combines in the Tafel step.^47^ In the present work, we show how this process can occur in greater detail for the Ni–Fe/NiFe(OH)_2_ system under hydrated conditions.

**Figure 7:**
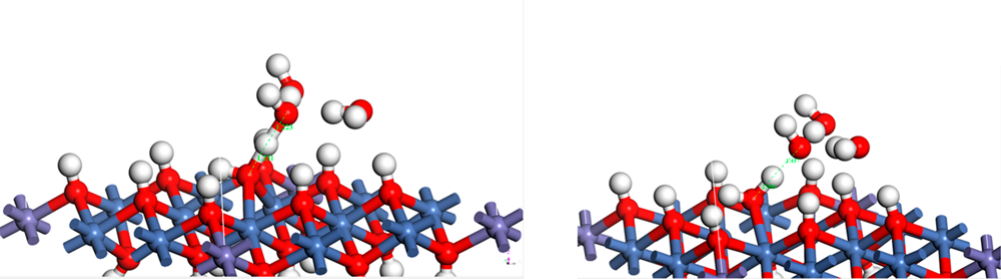
Proposed model for the transfer of a proton from a nonadsorbed water molecule to a surface OH to produce a strongly adsorbed water, which can subsequently transfer a proton to a metal cluster, as shown in Figure 6. Interestingly, water spontaneously adsorbs with a bond strength approaching that in water on this Ni_8/9_Fe_1/9_(OH)_2_ surface, whereas on Ni(OH)_2_, the approaching water only forms a hydrogen bond.

In the present case involving Ni(OH)_2_ sheets, all of the surface oxygens can be assumed to be protonated, so that an additional adsorbed H would essentially create a strongly adsorbed water molecule. Figure 7 shows that a water molecule in the liquid phase next to a Ni(OH)_2_ or Ni_8/9_Fe_1/9_(OH)_2_ surface can protonate a surface OH group, creating a strongly adsorbed water, with the production of an OH^–^ ion. This process is expected to have a low activation energy and thus not to be the rate-determining step. The activation energy would be smaller on the Ni_8/9_Fe_1/9_(OH)_2_ surface, with water spontaneously attaching to the surface, creating an interesting metastable state with equal O–H bond lengths.

As seen in Figure 7, the strongly adsorbed water molecule is raised somewhat from the plane formed by the other O atoms. Thus, it can desorb easily as a free water molecule, especially if there are defects in the surface, i.e., Ni or Fe vacancies. If the surface is ideal, there would be three metal–oxygen bonds holding the water molecule in place. With metal atom vacancies, there might only be one or two metal–oxygen bonds holding the water in place. We propose that this is the reason that the defective or disordered surface is not as active for the HER; i.e., there are insufficient strongly adsorbed water molecules available to transfer H atoms to the metal catalyst surface. In addition, it is considered that the ordered interface between Ni(OH)_2_ and NiFe can help to facilitate the transfer of the H^+^ from the former to the latter.

**Figure 8:**
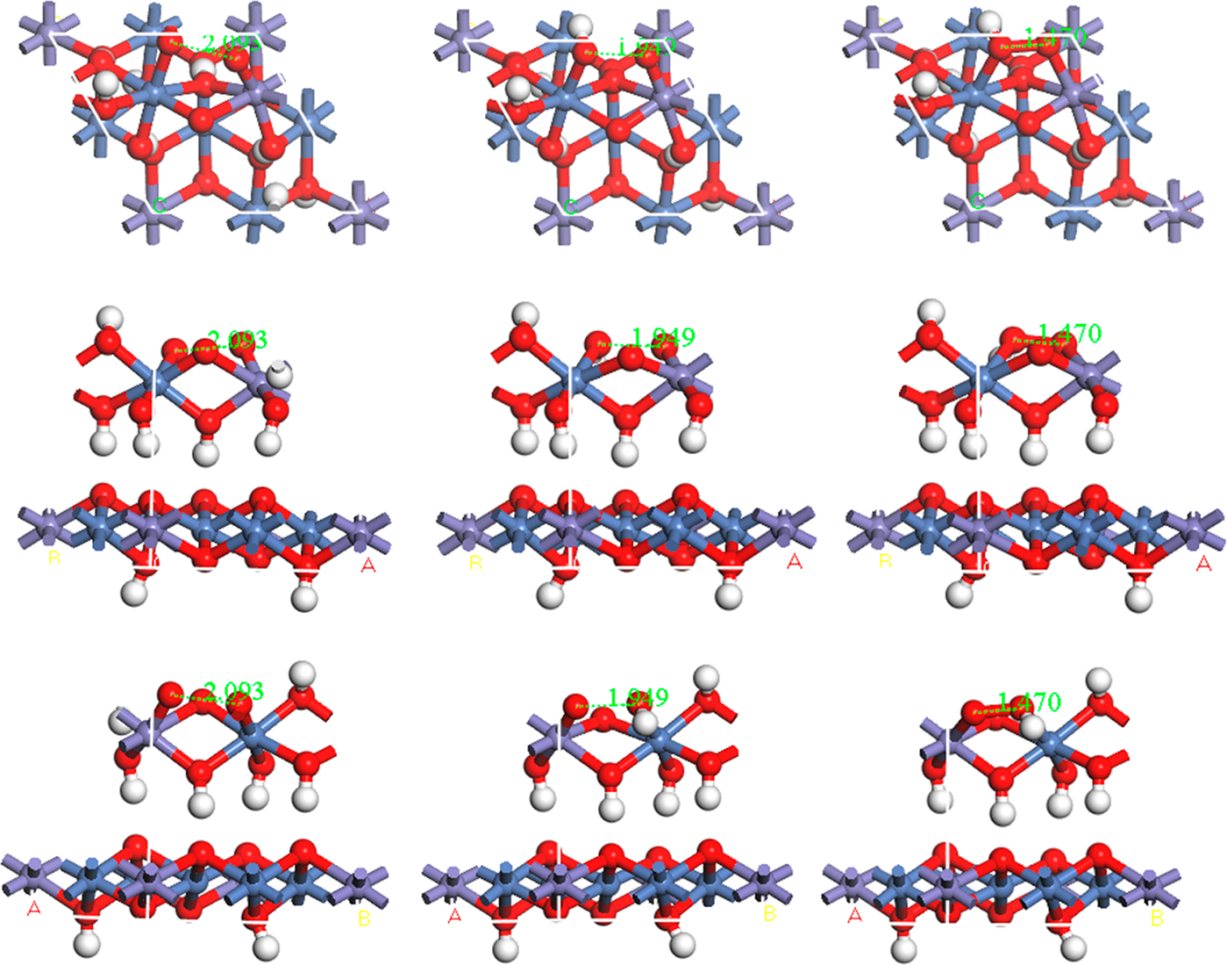
Proposed model for the OER-active surface, based on a NiOOH structure, with 1/4 of the Ni atoms being replaced with Fe. At left are shown top, side, and side (180° rotated) views of the initial structure, with an O–O bond length of 2.093 Å; in the middle is shown the activated state, with the two O atoms 1.949 Å apart; at right are shown the two O atoms in a relaxed configuration with an O–O bond of 1.470 Å.

To investigate the OER catalytic mechanism, we have taken the β-NiOOH structure and have partially substituted it with Fe. Then, we have removed half of the metal atoms in the top layer to create a row-type vacancy to simulate the amorphous NiFeOOH surface. As shown in Figure 8, interestingly, the alternating edge oxygen atoms neighboring the vacancy are tilted toward each other, with one being an OH. This pair of O atoms was found to have an extremely low activation energy, 0.042 eV, for the formation of O_2_. The overall energy gain in going from O–OH to O–OH was only −0.189 eV, i.e., slightly exothermic. Thus, the nearly barrierless reaction for O–O bond formation can explain the high OER activity present in amorphous Ni–Fe oxide catalysts.

## Conclusions

We have constructed amorphous/crystalline NiFe-based catalysts with alloy–oxide interfaces and investigated their electrocatalytic performance toward the HER and OER in alkaline solution. The crystalline Ni–Fe oxide demonstrated distinctive advantages over the amorphous one in facilitating the HER. In contrast, the amorphous or poorly crystalline oxide structure rendered a more favorable OER activity than that for the crystalline structure. Based on the DFT results, the crystalline Ni–Fe oxide was able to promote water dissociation on the Ni_8/9_Fe_1/9_(OH)_2_ surface and transfer a proton to the metal cluster, where it could be converted to an adsorbed H atom. The strongly adsorbed water molecule as a carrier of a proton that can be converted to H adsorbed on the Ni metal catalyst particle requires an ordered Ni(OH)_2_ surface to hold it in place and prevent it from desorbing, thereby facilitating the HER activity. In addition, an ordered interface is necessary for the transfer process. The amorphous Ni–Fe oxide with disordered structure helps lower the activation energy barrier for the formation of the OOH intermediate, which could be the origin of the remarkable OER activity. This work provides inspiration for catalyst optimization for targeted applications by tuning the crystallinity of heterostructured metal oxide materials.

## Supporting Information Available

Cyclic voltammograms, elemental compositions of catalyst materials, HRTEM image and FFT patterns before and after OER, ECSA-normalized OER polarization curves, and details of the DFT calculations (PDF)

## Notes

The Hydrogen and Fuel Cell Nanomaterials Center, University of Yamanashi, was previously named the Fuel Cell Nanomaterials Center.
