# Supplementary material for: NiFe Alloy Integrated with Amorphous/Crystalline NiFe Oxide as an Electrocatalyst for Alkaline Hydrogen and Oxygen Evolution Reactions
Source: ACS Omega. 2023 Mar 29;8(14):13068–77. doi: 10.1021/acsomega.3c00322 (PMC10099113; doi:10.1021/acsomega.3c00322)
Supplement: Supplementary file 1 — ao3c00322_si_001.pdf [file ao3c00322_si_001.pdf]

# Supporting Information

## NiFe Alloy Integrated with Amorphous/Crystalline NiFe Oxide as an Electrocatalyst for Alkaline Hydrogen and Oxygen Evolution Reactions

Guoyu Shi[§], Chisato Arata[//], Donald A. Tryk[§], Tetsuro Tano[§], Miho Yamaguchi[§], Akihiro Iiyama[§], Makoto Uchida[§], Kazuo Iida[//], Sumitaka Watanabe[//], Katsuyoshi Kakinuma*[,§]

[§]Hydrogen and Fuel Cell Nanomaterials Center, University of Yamanashi, Miyamae 6-43, Kofu 400-0021, Yamanashi, Japan

[//]R&D Center, Nihon Kagaku Sangyo Co., Ltd., Nakane 1-28-13, Soka, Saitama 340-0005, Japan

*Corresponding Author

Tel/Fax: +81-55-254-7143; E-mail: kkakinuma@yamanashi.ac.jp

**Table S1** Compositions of the catalysts determined from EDX mapping analysis.

| Catalyst | Atomic percentage, at. % | |
|---|---|---|
| | Ni | Fe |
| NiFeO-1 | 87.58 | 12.42 |
| NiFeO-2 | 87.60 | 12.40 |

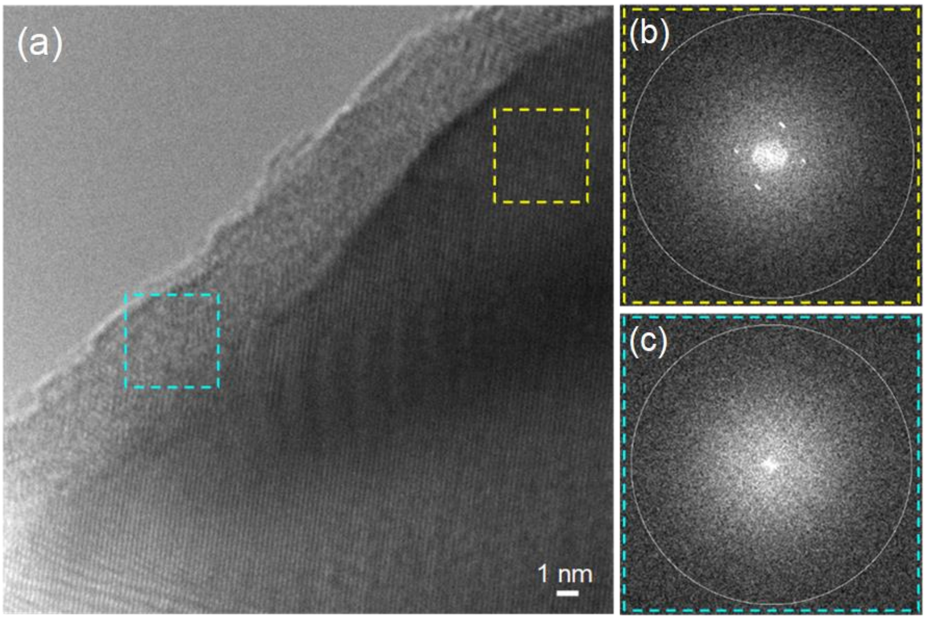


**Figure S1.** HRTEM image (a) and fast Fourier transformation (FFT) patterns of the selected regions marked by yellow and aqua green squares (b, c) for $Ni_{0.88}Fe_{0.12}O$-1 catalyst.

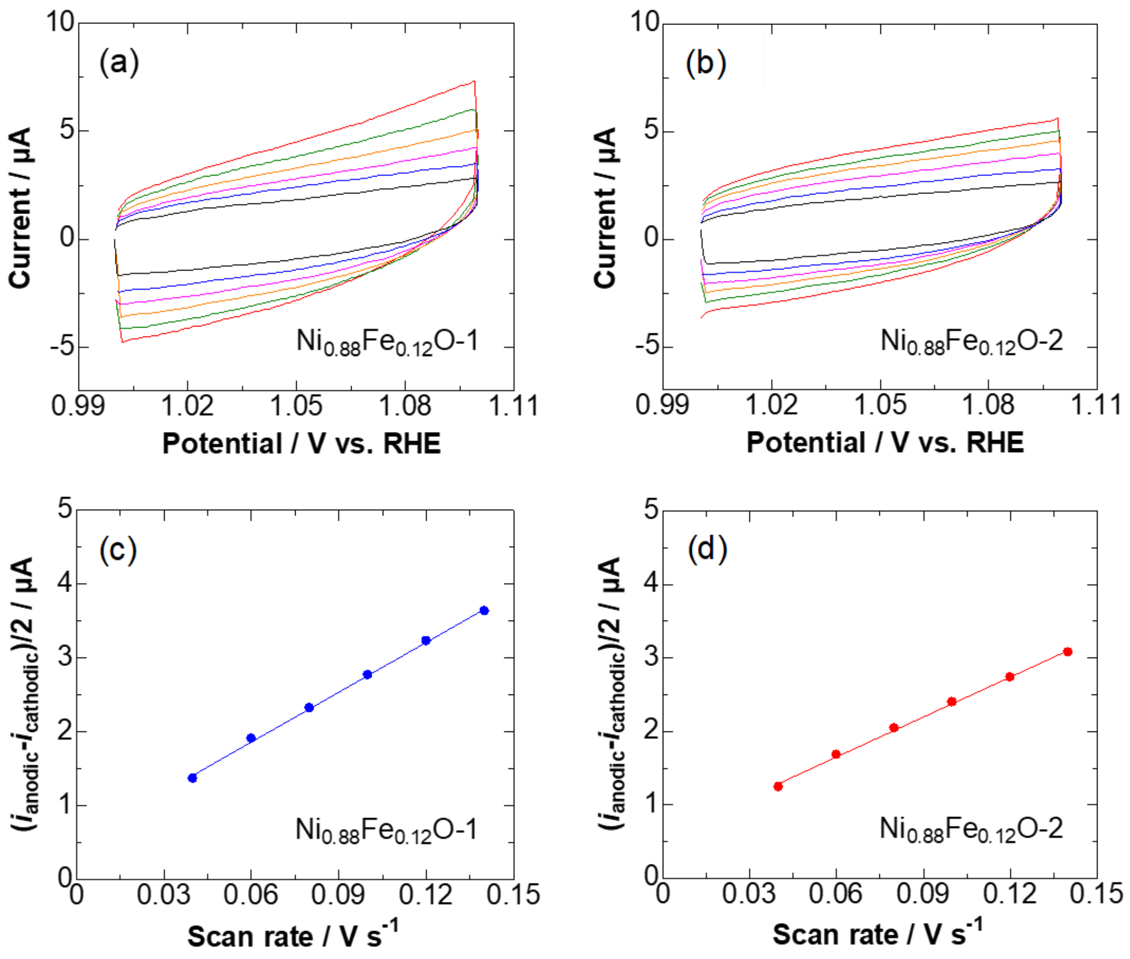


**Figure S2.** (a,b) Cyclic voltammograms of $Ni_{0.88}Fe_{0.12}O$-1 and $Ni_{0.88}Fe_{0.12}O$-2 measured in a non-Faradaic potential region at the following scan rate: (—) 0.04, (—) 0.06, (—) 0.08, (—) 0.10, (—) 0.12, and (—) 0.14 V $s^{-1}$. (c,d) Variation of double-layer charging currents (($j_{anodic}$-$j_{cathodic}$)/2) at 1.05 V with potential scan rate for $Ni_{0.88}Fe_{0.12}O$-1 and $Ni_{0.88}Fe_{0.12}O$-2. The ECSA was calculated by using the equation of ECSA = $C_{dl}/C_s$, where $C_s$ is the specific capacitance of the samples, and a value of 0.04 mF $cm^{-2}$ was adopted herein. The values of $C_{dl}$ (double-layer capacitance) were obtained from the slopes of the linear fits to the data in (c) and (d).

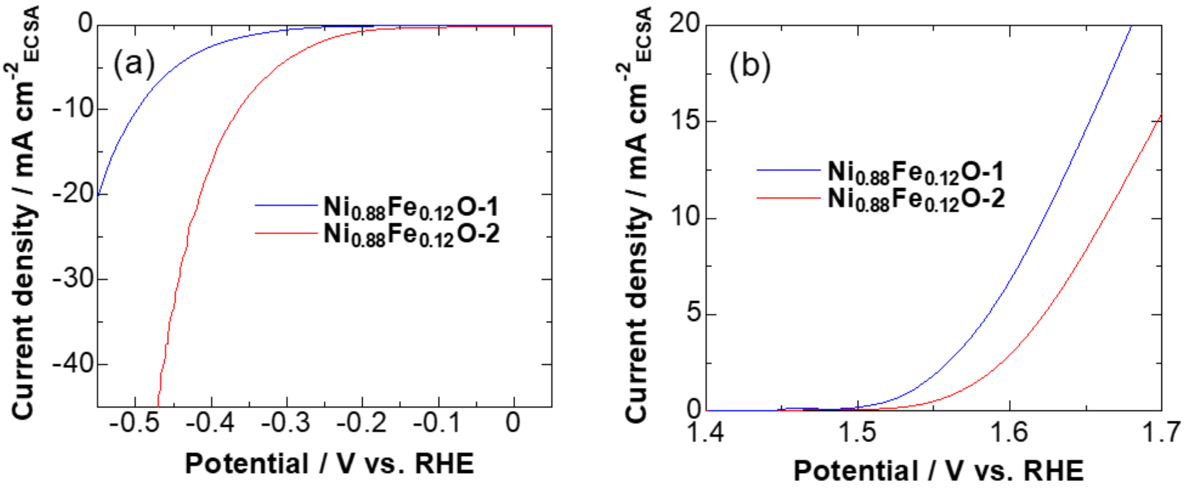


**Figure S3.** (a) HER and (b) OER polarization curves, in which the current was normalized by the ECSA of each catalyst.

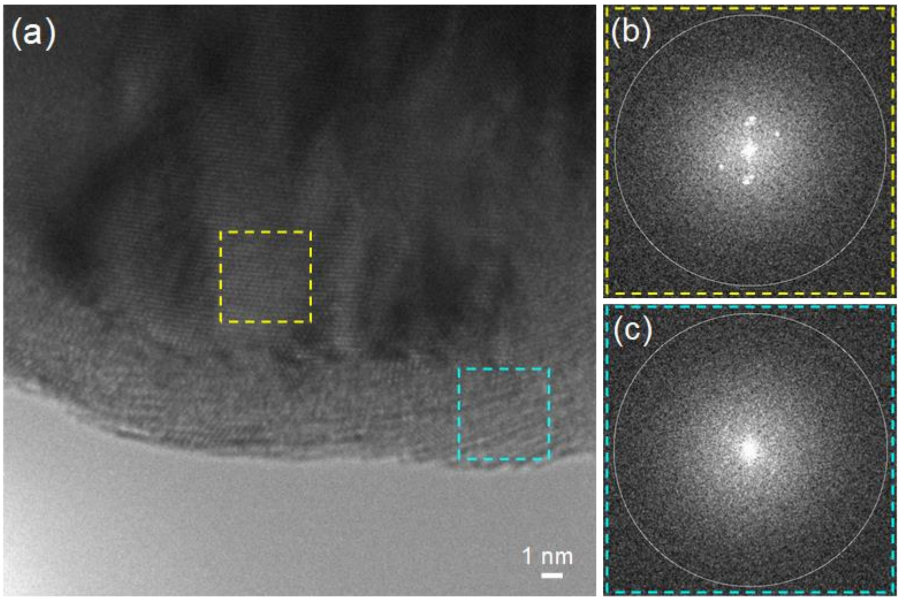


**Figure S4.** HRTEM image (a) and FFT patterns of the selected regions marked by yellow and aqua green squares (b, c) for $Ni_{0.88}Fe_{0.12}O$-1 catalyst after OER.

## DFT calculations

High-accuracy electronic structure density function theoretical (DFT) calculations were carried out by use of the $DMol^3$ package (BIOVIA, version 2021).[1] The geometric optimizations for periodic boundary conditions were carried out with the hardness-conserving semilocal pseudopotential[2] and the PBE functional.[3] The geometry optimizations were carried out with medium-quality settings (convergence criteria, $2 \times 10^{-5}$ Ha, maximum force 0.004 Ha/Å, maximum displacement 0.005 Å with a numeric quality basis set with polarization functions), with all-electron basis. To facilitate scf convergence, a minimum kinetic energy was applied to the electrons (thermal smearing) 0.0005 Ha, the latter being 1/10 of the usual value of 0.005 Ha. These low values were used in order to facilitate scf convergence as well as to avoid unwanted effects of excessive electron energy, as described by Basiuk et al.[4]

For the calculations involving the HER, a β-$Ni(OH)_2$ 3 × 3, 2-layer model was used to simulate the hydrated oxide.[5] In both layers, one of the 9 Ni was replaced with Fe. The metal atom xyz coordinates in the bottom layer were constrained, while, in the upper layer, only the x and y coordinates of the metal atoms were constrained. This was done in order to conserve computation time, since the interaction between the two layers is somewhat weak, so that the registry between the two could easily shift. This is expected to have a minimal impact on the results. To simulate the metal cluster on the surface, initially a large 44-atom Ni(110) cluster was used and place in a quasi-epitaxial position onn the $Ni(OH)_2$ surface. However, it proved to require excessive computation time, so this cluster was trimmed to only 8 Ni, and then one of these was replaced with Fe to approximate the Ni:Fe ratio used in the experiments. The surface of the metal particle was covered with a maximum of adsorbed H in order to simulate the conditions of the HER.

For the OER simulations, a β-NiOOH 2 × 2, 2-layer model was used.[6] To simulate the NiFe system, a single Ni was replaced with Fe in each layer, and for the disordered oxide, two of the metal atoms were removed in the upper layer, leaving one Ni and one Fe. As for the $Ni(OH)_2$ system, the coordinates of the lower layer metal atoms were constrained while only the x and y coordinates of those in the upper layer were constrained. The geometry optimizations were also carried out with medium setting and all-electron basis.

## References


(1) Delley, B. A Scattering Theoretic Approach to Scalar Relativistic Corrections on Bonding. *Int. J. Quant. Chem.* **1998**, *69*, 423-433.

(2) Delley, B. Hardness Conserving Semilocal Pseudopotentials. *Phys. Rev. B* **2002**, *66*, 155125-155133.

(3) Perdew, J. P.; Burke, K.; Ernzerhof, M. Generalized Gradient Approximation Made Simple. *Phys. Rev. Lett.* **1996**, *77*, 3865-3868.

(4) Basiuk, V. A.; Prezhdo, O. V.; Basiuk, E. V. Thermal Smearing in DFT Calculations: How Small Is Really Small? A Case of La and Lu Atoms Adsorbed on Graphene. *Mater. Today Commun.* **2020**, *25*, 101595.

(5) Enoki, T.; Tsujikawa, I. Magnetic Behaviours of a Random Magnet, $Ni_pMg_{(1-p)}(OH_2)$. *J. Phys. Soc. Jpn* **1975**, *39*, 317-323.

(6) Casas-Cabanas, M.; Canales-Vázquez, J.; Rodríguez-Carvajal, J.; Palacín, M. R. Deciphering the Structural Transformations during Nickel Oxyhydroxide Electrode Operation. *J. Am. Chem. Soc.* **2007**, *129*, 5840-5842.
